# Title-molecular diagnostics of dystrophinopathies in Sri Lanka towards phenotype predictions: an insight from a South Asian resource limited setting

**DOI:** 10.1186/s40001-023-01600-x

**Published:** 2024-01-09

**Authors:** Nalaka Wijekoon, Lakmal Gonawala, Pyara Ratnayake, Roshan Liyanage, Dhammika Amaratunga, Yetrib Hathout, Harry W. M. Steinbusch, Ashwin Dalal, Eric P. Hoffman, K. Ranil D. de Silva

**Affiliations:** 1 Interdisciplinary Center for Innovation in Biotechnology and Neuroscience, Faculty of Medical Sciences, University of Sri Jayewardenepura, Nugegoda, 10250 Sri Lanka; 2 Department of Cellular and Translational Neuroscience, School for Mental Health and Neuroscience, Faculty of Health, Medicine & Life Sciences, Maastricht University, 6200 Maastricht, The Netherlands; 3 Lady Ridgway Children’s Hospital, Colombo, 00800 Sri Lanka; 4 Princeton Data Analytics, Princeton, NJ 08544 USA; 5 School of Pharmacy and Pharmaceutical Sciences, Binghamton University, Binghamton, NY 13902 USA; 6 Diagnostics Division, Centre for DNA Fingerprinting and Diagnostics, Hyderabad, 500039 India; 7 Institute for Combinatorial Advanced Research and Education (KDU-CARE), General Sir John Kotelawala Defence University, Ratmalana, 10390 Sri Lanka

**Keywords:** Mutations, Dystrophin, South Asia, Muscular Dystrophies

## Abstract

**Background:**

The phenotype of Duchenne muscular dystrophy (DMD) and Becker muscular dystrophy (BMD) patients is determined by the type of *DMD* gene variation, its location, effect on reading frame, and its size. The primary objective of this investigation was to determine the frequency and distribution of DMD gene variants (deletions/duplications) in Sri Lanka through the utilization of a combined approach involving multiplex polymerase chain reaction (mPCR) followed by Multiplex Ligation Dependent Probe Amplification (MLPA) and compare to the international literature. The current consensus is that MLPA is a labor efficient yet expensive technique for identifying deletions and duplications in the *DMD* gene.

**Methodology:**

Genetic analysis was performed in a cohort of 236 clinically suspected pediatric and adult myopathy patients in Sri Lanka, using mPCR and MLPA. A comparative analysis was conducted between our findings and literature data.

**Results:**

In the entire patient cohort (*n* = 236), mPCR solely was able to identify deletions in the DMD gene in 131/236 patients (DMD-120, BMD-11). In the same cohort, MLPA confirmed deletions in 149/236 patients [DMD-138, BMD -11]. These findings suggest that mPCR has a detection rate of 95% (131/138) among all patients who received a diagnosis. The distal and proximal deletion hotspots for DMD were exons 45–55 and 6–15. Exon 45–60 identified as a novel in-frame variation hotspot. Exon 45–59 was a hotspot for BMD deletions. Comparisons with the international literature show significant variations observed in deletion and duplication frequencies in *DMD* gene across different populations.

**Conclusion:**

DMD gene deletions and duplications are concentrated in exons 45–55 and 2–20 respectively, which match global variation hotspots. Disparities in deletion and duplication frequencies were observed when comparing our data to other Asian and Western populations. Identified a 95% deletion detection rate for mPCR, making it a viable initial molecular diagnostic approach for low-resource countries where MLPA could be used to evaluate negative mPCR cases and cases with ambiguous mutation borders. Our findings may have important implications in the early identification of DMD with limited resources in Sri Lanka and to develop tailored molecular diagnostic algorithms that are regional and population specific and easily implemented in resource limited settings.

**Supplementary Information:**

The online version contains supplementary material available at 10.1186/s40001-023-01600-x.

## Background

Duchenne muscular dystrophy (DMD), OMIM #310,200 and Becker muscular dystrophy (BMD), OMIM #300,376 are X-linked recessive disorders caused by pathogenic variations in the *DMD* gene (OMIM *300,377, HGNC ID: 29). These conditions are collectively referred to as dystrophinopathies [1, 2]. The prevalence of DMD and BMD, according to a recent meta-analysis, is 4.8 per 100,000 and 1.6 per 100,000, respectively [3]. It is important to note that mutations in the *DMD* gene predominantly affect males. However, there have been reports indicating that a percentage ranging from 2.5% to 7.8% of females have also been affected by these mutations, thereby being classified as symptomatic carriers. [4].

Receiving an accurate diagnosis of dystrophinopathy is crucial to avoid the lengthy and somber diagnostic odyssey. Even though the average age of diagnosis for DMD remained over a decade as 5 years, it has been reported that the average age of diagnosis in Europe has decreased below the age of 3 years, reflecting the impact of enhanced access to molecular diagnostics and increased primary physician awareness [5]. In contrast, diagnostic delays in DMD persist with notable frequency within traditionally marginalized populations encompassing individuals hailing from developing nations and those of a lower socioeconomic stratum [6, 7].

It can be challenging to differentiate DMD from BMD at a younger age. In this case, the "reading frame rule" [8] can help aid differential diagnosis, where DMD patients typically show out-of-frame deletions, whereas BMD patients typically show in-frame deletions [9]. The frame-shift hypothesis can predict the occurrence of DMD in 90% of cases and BMD in 94% of instances, with about 10% of genetic variations not adhering to the reading frame rule [10]. Exceptions to the reading frame rule highlight the intricacy of the condition and show that factors other than the reading frame affect how the dystrophin protein is expressed. These factors include the type of variation, where it is located within the *DMD* gene, and its size [11]. When evaluating the results of a molecular diagnosis to characterize dystrophinopathies, this cumulative impact of the type, size and localization of the variation is of importance.

To the best our knowledge, the present study is the first and the largest comprehensive genetic analysis of a cohort of 236 clinically suspected pediatric and adult myopathy cohort in a geographically defined South Asian population; Sri Lanka, using a combined approach of Multiplex PCR (mPCR) and Multiplex Ligation Dependent Probe Amplification (MLPA). The aims of this study are: (i) to determine the frequency and distribution of DMD gene variants (deletions/duplications) in Sri Lanka through the utilization of a combined approach involving mPCR followed by MLPA and compare to the international literature and (ii) to determine the applicability of the "reading frame rule" in Sri Lankan DMD/BMD patient population.

## Methodology

### Patient recruitment

A total of 236 patients [Age range (Mean); 1.5–42 Yrs (9 Yrs); Gender (Male-233:Female-3)] exhibiting characteristic clinical findings of Muscular Dystrophy were enrolled in the study from 2014 to 2022. Clinical diagnosis was based on the diagnostic recommendations by Bushby et al. [12]. Sociodemographic characteristics and clinical data of the patients were documented using a standard questionnaire and clinical batteries that included North Star Ambulatory Assessment (NSAA), Vignos Scale, Brook Scale and Medical Research Council Scale (MRC). Three females [age-9 Yrs (family history of elevated CPK; 9596 U/L, and NSAA-27/34), 10 Yrs (elevated CPK; 6786 U/L, NSAA- 23/34, no family history of symptoms) and 16 Yrs (elevated CPK; 3725 U/L, wheel chair bound at 15 Yrs of age and no family history of symptoms)], were too enrolled to assess the symptomatic carrier status.

Recruitment was conducted through neurology clinics in various government hospitals across Sri Lanka's Western, North-Western, North Central, Central, Southern, and Northern Provinces, as well as through pro bono mobile clinics and home visits. These patients were referred to the Interdisciplinary Center for Innovation in Biotechnology and Neuroscience (ICIBN) of the University of Sri Jayewardenepura until 2020, and then to the Institute for Combinatorial Advanced Research and Education (KDU-CARE), General Sir John Kotelawala Defence University (KDU), Sri Lanka for genetic testing.

Every participant provided written informed consent, where applicable. The assent of a proxy was obtained for patients unable to provide their own. This study adheres to the ethical standards of Sri Lankan institutional review boards that follow the Helsinki Declaration (Ethical Approval Nos. 449/09 and 38/19 from The Ethics Review Committee, Faculty of Medical Sciences, University of Sri Jayewardenepura, and Ethical Approval No. LRH/D/06/2007 Lady Ridgeway Hospital for Children, Sri Lanka).

### Molecular Diagnostics

This study utilized the molecular diagnostic approach described in Wijekoon et al. [13] under the same corresponding author [13]. A summary of this approach is as follows. The initial diagnostic test for detecting deletions and duplications followed a level one testing approach, utilizing Multiplex PCR (mPCR) for 20 exons covering proximal and distal hot-spot regions of the *DMD* gene as described by Chamberlain et al. [14] and Beggs et al. [15] followed by the MLPA assay (MRC Holland SALSA MLPA Probe mixes P034 and P035) for all the clinically diagnosed dystrophinopathy patients. The diagnostic procedure was established utilizing the primary molecular diagnostic recommendations as outlined by Abbs et al. [17], as well as the revised edition by Fratter et al. [2], in alignment with the European Molecular Quality Genetics Network's (EMQN) optimal practice guidelines for genetic testing in dystrophinopathies [2, 16, 17]. To ascertain the impact of variations on the reading frame, the frame-shift checker available on the Leiden Muscular Dystrophy website (www.dmd.nl) was utilized to scrutinize all identified deletions and duplications where the number of patients who are following and not following the reading frame rule was identified. The comparative effectiveness of mPCR and MLPA was assessed by examining the individual capabilities of each method in detecting deletions and deletion boundaries of the *DMD* gene in genetically confirmed patients.

### Comparative analysis with existing literature data from various countries representing diverse geographical regions

A literature review was conducted to compare the findings of this study, including the percentages of DMD gene deletion/duplication and the mean age of confirmatory molecular diagnosis, with existing literature data. The following method was employed for the literature review. The review process was structured into three primary stages: title screening, abstract screening, and document screening. A comprehensive search was conducted in globally recognized databases such as PubMed, Medline, Scopus, Embase, and Springer to identify relevant literature. The search was conducted using a combination of key words: Duchenne Muscular Dystrophy, Becker Muscular Dystrophy, Mutation pattern, MLPA, and Diagnostic delay. A total of 861 publications were identified. All titles underwent a screening process, resulting in the selection of 275 documents for abstract screening. A total of 275 abstracts were reviewed, and 120 articles were identified as potentially meeting the inclusion criteria related to Duchenne Muscular Dystrophy, Becker Muscular Dystrophy, MLPA, Mutation pattern, and Diagnostic delay. Ultimately, a comprehensive evaluation was conducted on the complete text of all 120 documents that were retained. This evaluation adhered to the same set of criteria for inclusion and exclusion as the initial screening of abstracts. As a result, a total of 49 papers were deemed suitable for inclusion in the subsequent comparative analysis.

### Data analysis

Our findings on *DMD* gene variation types, hotspot locations, and deletion/duplication percentages, age at molecular diagnosis were compared with available data in literature from countries representing different geographical regions that have utilized the same molecular diagnostic protocol as our study. To test whether geographical region of various patient populations has an effect on the deletion/duplication percentages, the available country-specific data from the literature were graphically analyzed using boxplots followed by an ANOVA test. Those declared significantly different by ANOVA (*p* < 0.05) were then also studied using Tukey’s pairwise comparisons test. A comparative analysis was conducted to examine the mean age of confirmatory molecular diagnosis of DMD across countries representing Low and Middle-Income levels, as compared to countries representing High-Income levels. This analysis involved the use of boxplots to graphically represent the data. To ensure statistical power, the analysis categorized countries into two groups: Low and Middle-Income countries, and High-Income countries. This was necessary due to the limited availability of data on the mean age of confirmatory molecular diagnosis of DMD in only a few countries. Statistical analysis was performed using R Statistical software version 4.2.

## Results

### Demographic Characteristics of patient cohort

A total of 236 patients [Age range (Mean); 1.5–42 Yrs (9 Yrs); Gender (Male-233:Female-3)] exhibiting characteristic clinical findings of Muscular Dystrophy (Clinically diagnosed DMD-215, Clinically diagnosed BMD-21) were subjected to *DMD* gene deletion/ duplication analysis by mPCR and MLPA. Table 1 is a summary of demographic characteristics of the patient cohort.

**Table 1:** Demographic characteristics of the patient cohort

Characteristic	Considering MLPA based molecular diagnostic performed cases (*n* = 236)		
	DMD (*n* = 138)	BMD (*n* = 11)	Negative for MLPA (*n* = 87)
Age range (Mean)	1.5–18 Yrs (8 Yrs)	12–37 Yrs (21 Yrs)	8–42 Yrs (16 Yrs)
Age of onset range (Mean) (In our cohort, at the onset patients have received their first clinical evaluation)	1–8 Yrs (4 Yrs)	11–15 Yrs (13 Yrs)	7–18 Yrs (12 Yrs)
Gender	Male-138: Female-0	Male-11: Female-0	Male-84: Female-3
Frequent First neurological event at onset	Difficulty in climbing stairs- 49/138 (36%) and Frequent falling- 39/138 (28%)	Calf pain- 3/11 (27%) Difficulty in climbing stairs- 3/11 (27%) and Frequent falling- 3/11 (27%)	Difficulty in climbing stairs- 40/87 (46%) Frequent falling- 22/87 (25%)
Presence of development delay (Most patients were present with a combination of developmental delays)	76/138 (55%) Of which; Motor delay- 67/76 (88%) Language delay- 57/76 (75%) Vision & fine motor delay- 30/76 (39%)	No	4/87 (5%) Of which; Motor delay- 3/4 (75%) Language delay- 2/4 (50%)
NSAA mean	14/34	21/34	23/34
CPK Range	1670–45000 U/L	916–11,340 U/L	840–9750 U/L
Mean CPK	15,143 U/L*	4000 U/L*	4330

^*^p < 0.01

### Utility of mPCR and MLPA in the molecular diagnostics of the patient cohort

In the entire patient cohort (*n* = 236), mPCR solely was able to identify deletions in the DMD gene in 131/236 patients (DMD-120, BMD-11). In the same cohort, MLPA confirmed deletions in 149/236 patients [DMD-138, BMD -11]. Importantly deletion boundaries could be accurately detected by mPCR in a total of 100/236 (42%) patients. These findings suggest that mPCR has a detection rate of 95% (131/138) among all patients who received a diagnosis. Eighteen additional cases (18/236- 7.6%) (Detetions-5, Duplications-13) could be genetically diagnosed by MLPA over mPCR. The remaining 87 patients (37%) were negative for MLPA. Table 2 provides a summary of molecular diagnostic results achieved by mPCR and MLPA. Additional file 1: Table S1 summarizes the additional mutations and deletion borders identified by MLPA over mPCR (see Additional file 1: Table S1).

**Table 2:** Spectrums of deletions/duplications in dystrophin gene in our cohort

Type of Variation	Considering overall muscular dystrophy cohort (*n* = 236)	
Deletions by MLPA	136/236 (57.6%)	
Duplication by MLPA	13/236 (5.5%)	
Negative for mPCR	105/236 (44.5%)	
Negative for MLPA	87/236 (36.8%)#	
Mutation detection Percentage by mPCR	131/236 (55.5%)	
Precise diagnosis by mPCR (Exact deletion boundaries accurately detected by mPCR)	100/236 (42.4%)	
Mutation detection Percentage by MLPA	149/236 (63.1%)	
Additional Cases detected by MLPA	18/236 (7.6%)	
Type of Variation	Considering genetically confirmed cases (n = 149)	
	DMD (*n* = 138)	BMD (*n* = 11)
Deletions	125/138 (90.6%)	11 (100%)
Distal Deletions	92/125 (73.6%)	11 (100%)
Proximal Deletions	33/125 (26.4%)	Not identified
Variation hotspot for deletions	(exon 45–55) and (exon 6–15)	(exon 45–49)
Duplications	13/138 (9.4%)	Not identified
Distal Duplications	5/13 (38.5%)	Not identified
Proximal Duplications	8/13 (61.5%)	Not identified
Variation hotspot for duplications	(exon 6–10)	Not identified
Single exon deletions	28/138 (20.3%)	Not identified
Frequently deleted single exon	Exon 44−8/28 (28.6%) Exon 51- 7/28 (25%)	N/A
Single exon duplications	5/138 (3.6%)	Not identified
Frequently duplicated single exon	Exon 6- 2/5 (40%) Exon 49- 2/5 (40%)	N/A
Non-contiguous deletions	2/138 (1.4%)	Not identified
Non-contiguous duplications	2/138 (1.4%)	Not identified
All Out of frame variations	117/138 (84.7%) (Deletions and duplications out of frame)	0
All In frame variations	17/138 (12.3%) (Deletions and duplications in frame)	11 (100%) (In frame deletions only. Duplications not identified)
In frame variation hotspot for DMD	(Exon 45–60)	N/A
Deletions following reading frame rule	110/117 (94.0%) (Out of frame deletions)	11 (100%) (In frame deletions)
Deletions not following reading frame rule	13/17 (76.5%) (In frame deletions)	0 (Out of frame deletions)
Duplications following reading frame rule	7/117 (5.9%) (Out of frame duplications)	Not identified
Duplications not following reading frame rule	4/17 (23.5%) (In frame duplications)	Not identified
Development delay present with the DMD cases not following reading frame rule	Global development delay—15/17 (88.2%) Of which, Language delay—11/15 (73.3%) Motor development delay- 13/15 (86.7%)	N/A
Familial cases	21/138 (15.2%)	4/11 (36%)
Familial cases with deletions	20/21 (95.2%)	4 (100%)
Familial cases with duplications	1/21 (4.8%)	0
Patients eligible for available exon skipping gene therapy	Total 82/138 (59.4%) Exon 51 skipping- 30/82 (36.6%) Exon 53 skipping- 19/82 (23.2%) Exon 45 skipping- 19/82 (23.2%) Exon 50 skipping- 9/82 (10.9%) Exon 44 skipping- 5/82 (6.1%)	N/A

^#^The 3 females tested were also negative for *DMD* gene variant analysis by MLPA

### DMD gene deletions and duplications patterns in the Sri Lankan cohort

**Fig. 1:**
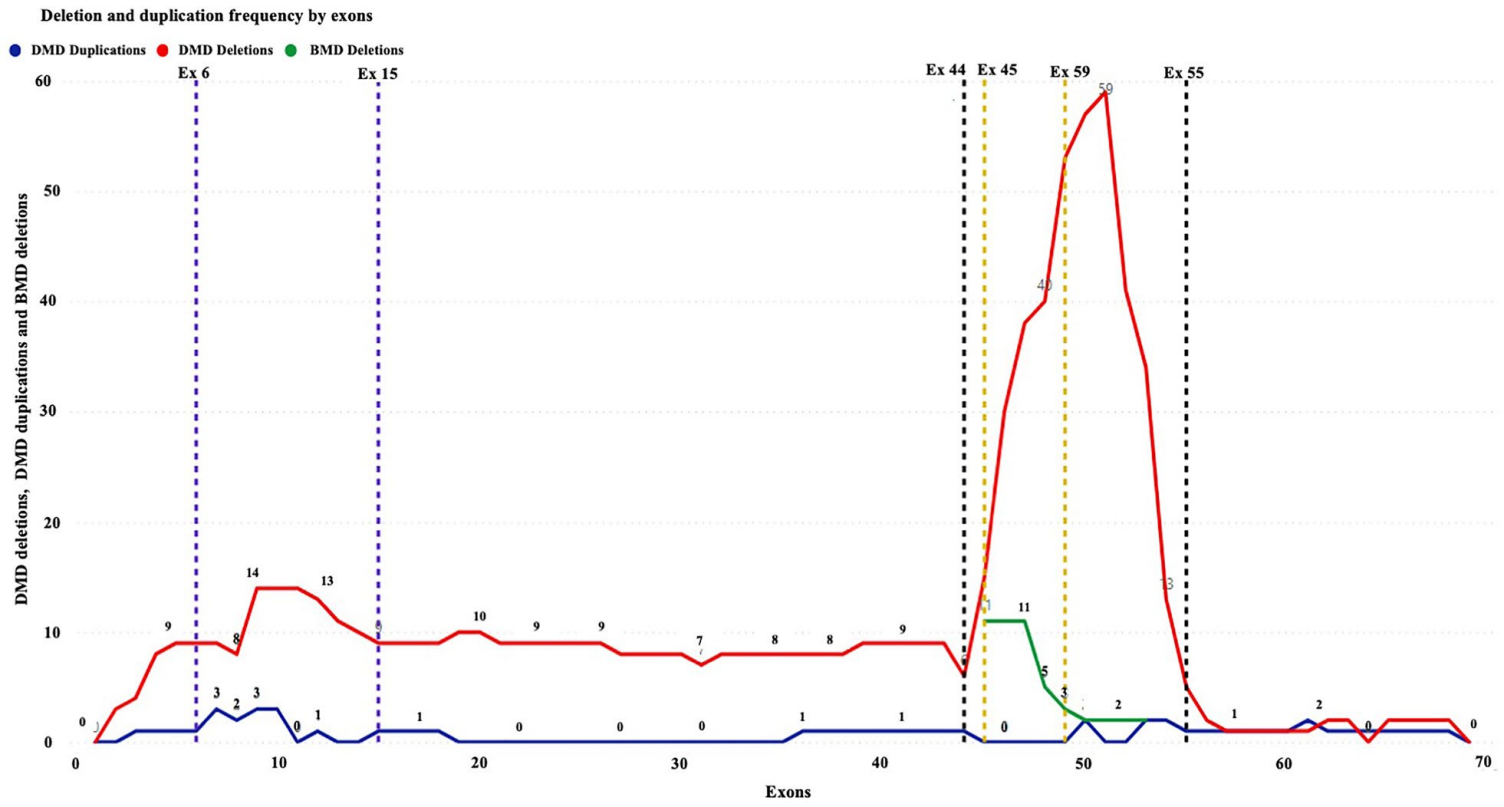
The deletion and duplication frequency by exons representing the clustering of deletion and duplication mutations in Sri Lankan dystrophinopathy patients. Clustering of deletion mutations in the exon 45–55 and 6–15 regions of the *DMD* gene and clustering of duplications in the exon 6–10. Red line represents DMD deletions, Blue line represents DMD duplications and the Green line represents BMD deletions

Table 2 provides a summary of the deletion and duplication variations, and their locations within the *DMD* gene. We observed clustering of deletion mutations in the exon 45–55 and 6–15 regions of the *DMD* gene and clustering of duplications in the exon 6–10 in our patient population (Fig. 1).

### Comparative analysis of DMD gene deletion and duplication locations, percentages and the mean age of confirmatory molecular diagnosis, with existing literature data

**Fig. 2:**
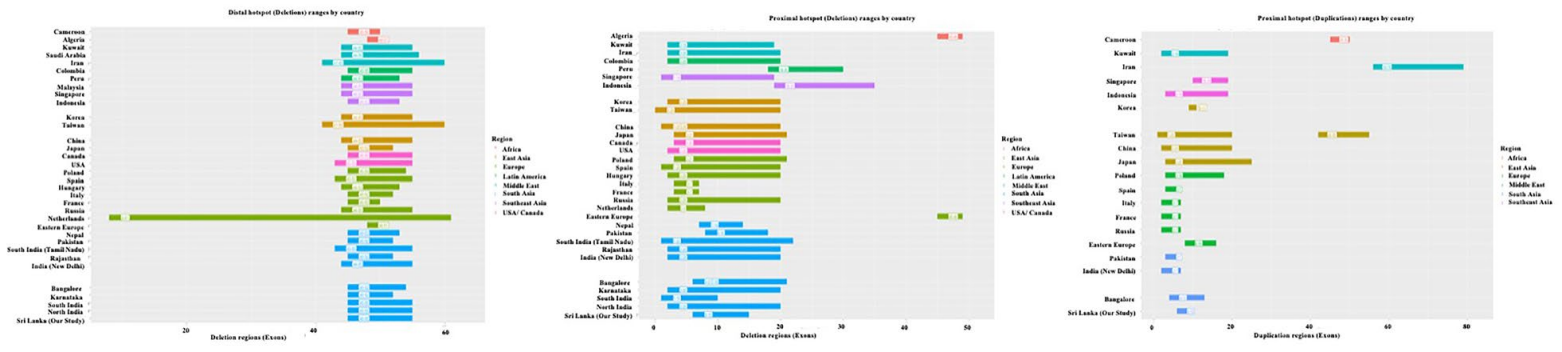
Comparative analysis of variability of *DMD* gene deletion and duplication hotspot location in different populations

We compared the variation hotspots of our cohort to the information available in the literature, as shown in Fig. 2. It was clear that South Asians represented a similar distal variation hotspot spanning exon 45–56 with the exception of the Netherlands, wherein the variations ranged from (exon 8–61). Exon 45–56 variation hotspot was consistent with the distal variation hotspots of the nations in South East Asia, East Asia, Europe, the USA–Canada, Latin America, the Middle East, and Africa. Furthermore, Peru, a country in Latin America, and Indonesia, a country in South East Asia, both had distinctive proximal hotspots ranging from (exon 18–30) and (exon 19–35), respectively. The proximal hotspots of Eastern European countries were notably spanning from (exon 45–49).

Although it was reported that duplications could occur at random anywhere in the *DMD* gene, the comparative analysis allowed us to determine that in the majority of populations, duplications are concentrated in exons between (exon 2–20). For duplications ranging from (exon 50–79), (exon 42–55), and (exon 45–50), respectively, Iran from the Middle East, Taiwan from East Asia and, the African region stood out as unique clusters of duplications.

**Fig. 3:**
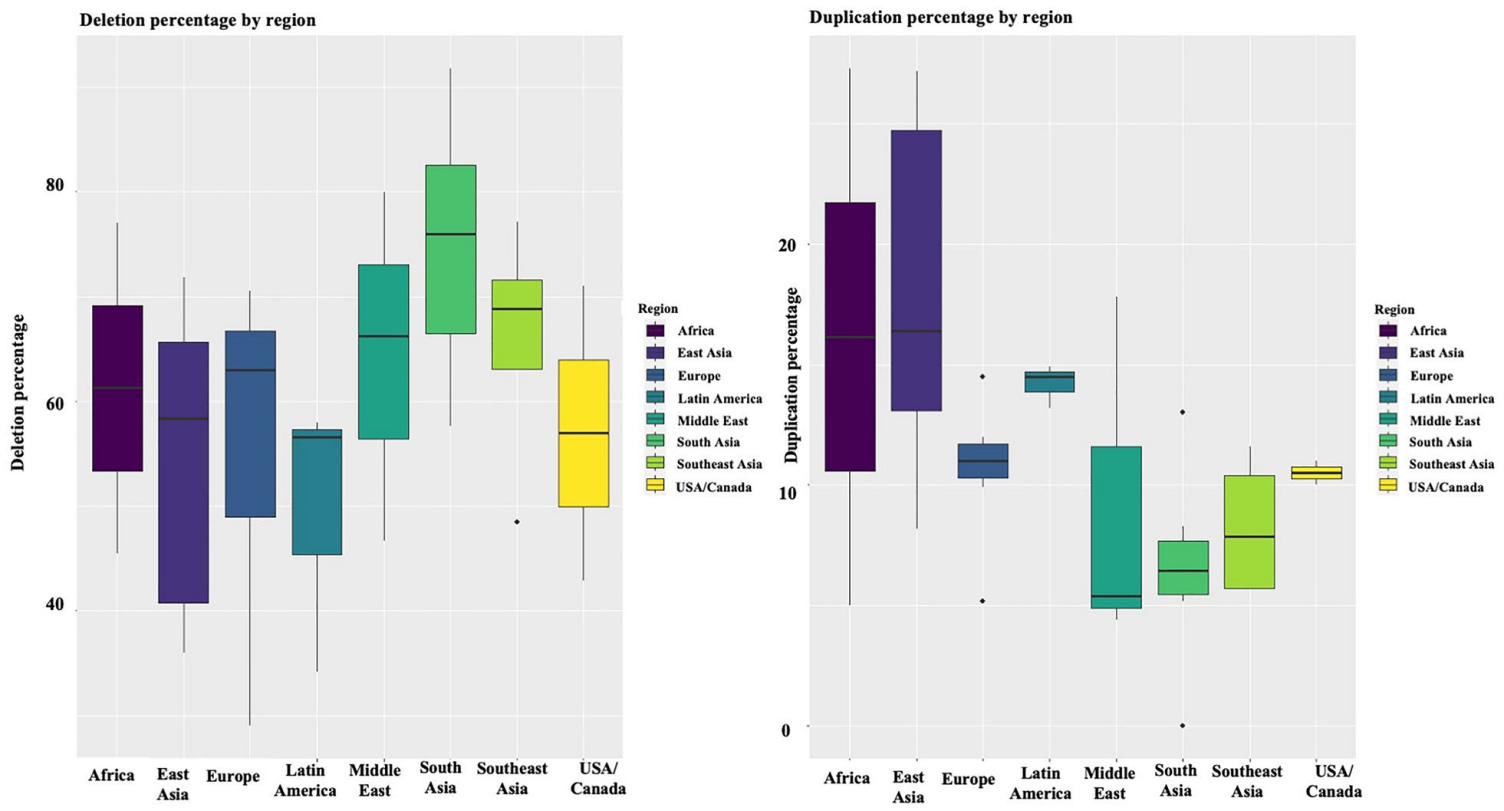
Data on country-specific percentages of duplications and deletions of the *DMD* gene from published literature were graphically evaluated for variability using boxplots

When the deletion and duplication percentages of studies from various geographical regions are compared, it was evident that the duplication percentages were significantly different (*p* < 0.05) among populations in South Asia Vs East Asia and South East Asia Vs East Asia. This is illustrated graphically in Fig. 3. The percentages of deletion, however, were not significantly different among populations in different geographical regions where South Asia Vs East Asia (*p* = 0.06) and South Asia Vs Europe (*p* = 0.06) were showing trends.

**Fig. 4:**
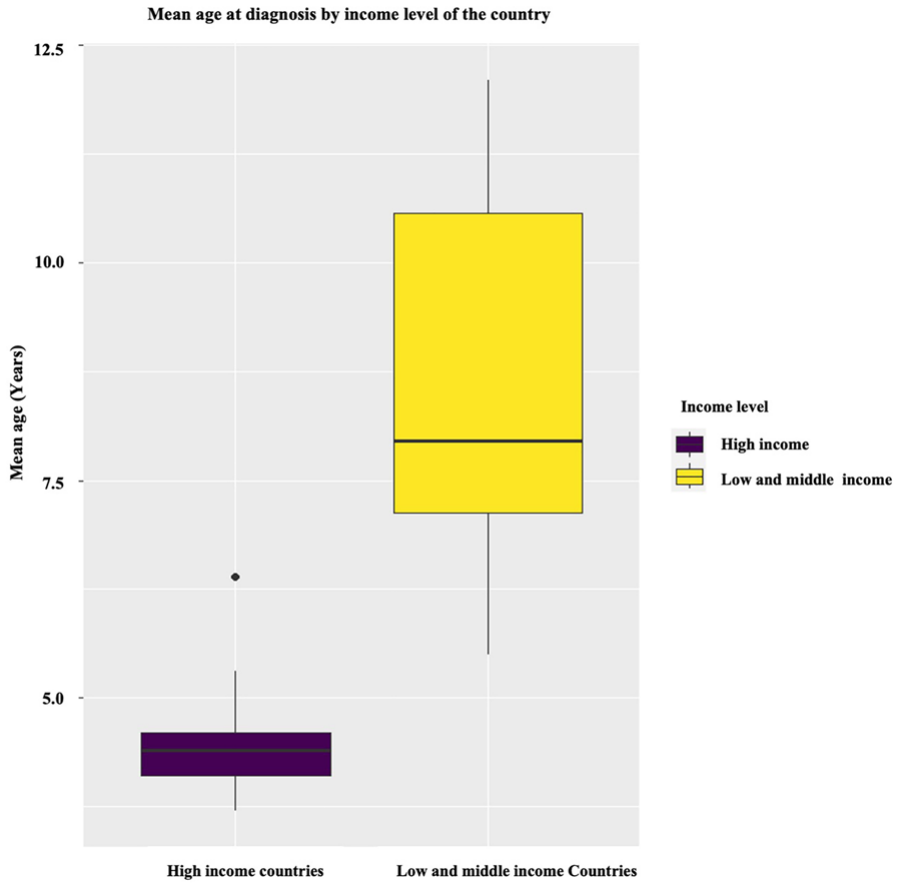
The average age of confirmatory molecular diagnosis of DMD between Low and Middle-Income countries and High-Income countries, *p* = 0.001

A comparative analysis was conducted on the mean age of confirmatory molecular diagnosis of DMD across countries representing Low and Middle-Income levels, namely Sri Lanka (our study), India [18], Thailand [19], Iran [20], Nepal [21], and Africa [22], versus to countries representing High-Income levels, including the USA [23], Eastern Europe, and Western Europe(5), based on the available literature. A significant difference (*p* = 0.001) was observed in the average age of confirmatory molecular diagnosis of DMD between Low and Middle-Income countries and High-Income Countries, as illustrated in Fig. 4.

### The applicability of the "reading frame rule" in Sri Lankan DMD/BMD patient population

Upon determining the impact of variations in the DMD gene on the reading frame in our population, it was observed that 117/138 (84.7%) DMD cases were attributed to out of frame variations, while 17/138 (12.3%) exhibited in frame variations. The observed hotspot for in-frame variation for DMD within our population was identified as Exon 45–60. Interestingly, it was observed that 15/17 (88.2%) that did not adhere to the reading frame rule were associated with global developmental delay. This is described in Table 2.

## Discussion

Based on our current understanding, this is the first and the largest study to use both mPCR and MLPA to conduct a genetic analysis on a cohort of 236 clinically suspected pediatric and adult myopathy patients in Sri Lanka (Table 1).

### Utility of mPCR and MLPA in molecular diagnostics

The utilization of MLPA is presently regarded as a labor-efficient primary method for detecting deletions and duplications of single or multiple exons in the *DMD* gene, as approximately 70% of dystrophinopathy patients exhibit such genetic alterations [2]. Despite not being the primary molecular diagnostic method in developed nations, mPCR remains a viable and economical option for detecting deletions, and is, therefore, utilized in many laboratories situated in countries with limited resources [24]. In our study as represented in Table 2 and Supplementary Table 01, mPCR could provide molecular diagnosis for 55% (131/236) of the patients, of which 76% (100/136) of the patients the exact deletion boundaries accurately detected by mPCR. In line with our findings, studies conducted in South India [25] and North India [26] identified (103/150) 68% and (161/217) 74%, respectively, as the mutation detection Percentage by mPCR. Intriguingly in our study 95% (131/138) of the patients with deletion mutations could be diagnosed by mPCR. In line with our findings, Nouri et al., [27] identified a deletion detection rate of 95% for mPCR in an Iranian population.

On average, the cost of MLPA is estimated to be five times higher than that of mPCR. The utilization of MLPA as the principal screening technique within the framework of a developing nation would involve considerable costs. Hence, the proposed approach of employing mPCR as the primary step, followed by MLPA, is a prudent and precise method to efficiently proceed with the genetic diagnosis of DMD in settings with limited resources as previously described by Murugan et al. for South India [25].

Using MLPA diagnostics (Table 2), we were able to identify 82/138 (60%) patients as amenable to available exon skipping therapies. Interestingly, the majority of our patients were eligible for exon 51 skipping (30/82), followed by exon 53 skipping (19/82) and exon 45 skipping (19/82), which is consistent with data reported in the South Asian region, including North India [28], Tamil Nadu [29], and Pakistan[30]. The authors extensively discussed the identification of DMD patients who can benefit from exon skipping therapies in a previous study by Wijekoon et al. in 2023. Therefore, this paper does not extensively discuss this topic [13].

Table 3 presents our findings of newly identified DMD variants in our study population, which have not been previously documented in the Leiden Muscular Dystrophy Gene Variant Database. According to reports, the introduction of genetic material from distinct geographical populations has the potential to augment genetic diversity and potentially engender novel genotypic configurations within populations that are not isolated or indigenous [96]. The island of Sri Lanka, situated at the southernmost tip of South Asia and along the proposed Southern migration route, has been inhabited by diverse ethnic groups such as Portuguese, Dutch, British, and Arabs. This presents an interesting opportunity to gain a distinct perspective on the initial settlement of the subcontinent [97, 98]. In this context, we can hypothesize that the genetic admixtures that have taken place in Sri Lanka may have contributed to the emergence of the novel DMD variants that have been detected in our population.

**Table 3:** Novel Variations identified in this study by MLPA [*Not previously reported in Leiden Muscular Dystrophy Gene Variant Database* (1)]

Sample Number	Case ID	Variation	In-frame/ Out-of- frame	Fragment mutated	Remarks
01	DMD050	Exon 52–67 duplicated	In-frame	c.7543-?_9807 + ?dup	A 4-year-old who was initially suspected of having Limb Girdle Muscular Dystrophy (LGMD) based on clinical phenotype was genetically confirmed to have DMD through our investigation. Full Scale IQ (FSIQ) of the patient was 42 (extremely low). Mother was a carrier for similar in-frame duplication spanning exons 52 to 67
02	DMD086	Exon 35–43 duplicated	Out-of-frame	c.4846-?_6290 + ?dup	A 5-year-old who was initially suspected of having Pompe disease (type II glycogen storage disease) was genetically confirmed to have DMD through our study. Short stature and delayed gross motor development accompanied by elevated levels of AST (278 U/L), ALT (284.5 U/L), and CPK (23,000 U/L). The activity of alpha-glucosidase was normal (0.312). The EMG concluded that there were no signs of myopathy
03	DMD066	Exon 1–42 deleted	Effect is difficult to predict	c.-244_6117 + ?del	Two siblings whose FSIQ was 85 and 94, respectively. Additionally, one sibling with the identical variation was reported to have a motor development delay and scored FSIQ-85, but the other sibling did not. Both individuals became wheelchair-bound at the age of 11 years
04	DMD067				
05	DMD109	Exon 2–29 deleted	Out-of- frame	c.32-?_4071 + ?del	Familial Case with an affected sibling. Nonetheless, the carrier status of the mother was not assessed as a result of lack of consent

Considering there have been reports of phenotypic differences in individuals carrying the same variation [11], healthcare practitioners should be more cautious when interpreting genomic results in the clinical environment for patients with similar variations and familial cases. Since distinct dystrophin-expressing tissues and cells may behave differently to specific defective dystrophins, such diversity is more common in BMD patients [33]. Furthermore, it is important to highlight that age can be confounded with phenotypic diversity when comparing patients with the same variation or familial cases. Therefore, it is advisable to take into account clinical batteries that are adjusted for age, such as the Weschler Intelligence Scale (WISC-IV). In this regard [99] have conducted a comprehensive analysis discussing the relationship between serum proteomics profile, cognitive assessment using WISC scores, and DMD gene mutations.

In this scenario, we have siblings in our patient cohort who have the same variation (out-of-frame deletion at exon 1–42), and whose Full-Scale IQ (FSIQ) as measured by the WISC-IV is 85 and 94, respectively. Additionally, one sibling with the identical variation was reported to have a motor development delay and scored FSIQ-85, but the other sibling did not (Table 3). Furthermore, the WISC-IV scores of another two distinct patients with identical variation (out-of-frame deletion at exon 45–52) were 83 and 67 for FSIQ, respectively. It's interesting that, despite the fact that this variation (deletion at exon 45–52) is likely to impact how brain dystrophin isoform Dp140 is expressed, only one patient outperformed the other on the WISC, indicating an intellectual deficit. These will serve as evidence of how complex it is to interpret genetic data in a clinical setting.

BMD patients with *DMD* variants in exon 1–8 and exon 41–45 impacting the Actin Binding Domain (ABD) and R16/R17 nNOS-binding domains are said to have a more severe presentation of BMD [1, 100]. We were unable to thoroughly evaluate this claimed connection due to the accumulation of *DMD* variants exclusively from exon 45–49 in our BMD cohort. Additionally, there have been reports on the comorbidity of Moyamoya disease [101], Frontometaphyseal Dysplasia [102], and Rippling muscle disease [103] with DMD. These findings highlight the intricacy of assessing the phenotypes of patients with dystrophinopathy in a clinical setting and underscore the need for the implementation of whole exome sequencing (WES) in the evaluation of dystrophinopathy patients exhibiting complex phenotypes and negative results on MLPA testing.

### Deletion, duplication percentages and their location in the DMD gene

Accordingly as summarized in Table 2, 58% (136/236) of the cases in our sample were due to deletions as analyzed by MLPA [DMD- 90% (125/138) and BMD- 100% (11/11)], and 6% (13/236) of the cases (all DMD) were due to duplications. The *DMD* gene has a higher degree of allelic heterogeneity compared to many genes, due to the spontaneous mutation rate and large size, with 79 exons spanning 2.2 Mb, and hot spots for deletion mutations. One or more exons are deleted in 60–65% of DMD patients and 85% of BMD patients, respectively [8, 45].

Data from the literature demonstrate that deletion and duplication percentages vary across various populations. When deletion percentages for various ethnicities are taken into account (Fig. 3), East Asians; Japan (61%) [46], Taiwan (36%) [47], China (58% and 71%) [48, 49] and Korea (46% and 72%) [50, 51], demonstrate a trend of lower deletion percentages in the *DMD* gene compared to South Asians (p = 0.06); Sri Lanka (90%), pan India (73%-91%) [52], [34, 53, 54], and Pakistan (87%) [30] and European countries; Netherlands (63%) [55], Italy (65%) [56], Spain (71%) [38], Hungary (67%) [80], Poland (61%) [81], Russia 49% [82] and France (67%) [36]. However, Algeria; a Northern African county has a deletion percentage of 77% [57]. In contrary, a study by Selvaciti et al. [57] on an overall group of 258 patients from Eastern European countries (Bosnia, Bulgaria, Croatia, Hungary, Lithuania, Poland, Rumania, Serbia, Ukraine and Cyprus) identified a lower deletion percentage (27%) (Fig. 3).

Elhavary et al. [58] proposed that during Ancient Islamic times, Muslim immigration from the Levant and Africa, coupled with intermarriage, contributed to the reinforcement of gene flow of the DMD gene among the Saudi population. Turkey; a Middle Eastern country crossroads between Europe and Asia, has been found to exhibit a complex genetic makeup resulting from admixture with populations from the Balkans, Caucasus, Middle East, and Europe. Notably, genetic analyses have revealed a closer genetic affinity of the Turkish population to Europeans [59]. In this context, findings of two studies conducted by Cavdarli et al. [60] and Ulgenalp et al. [106] reported a deletion percentage of 92.4% and 63.7% in the Turkish population, respectively [60, 61]. These percentages were observed to be higher than the deletion percentage reported in the Saudi population, which was 46.3%. According to Elhavary et al. 2019, the higher percentage of deletions observed in Turkey compared to Saudi Arabia may be attributed to the admixture of Turkish populations with those of European descent [58]. However, a study conducted by Toksoy et al. 2019 on Turkish patients with DMD report a deletion percentage of 48.8%, which is similar to the percentage observed in the Saudi community [62]. Therefore, it is crucial to conduct further investigations on the hypothesis that European admixture results in higher deletion percentages. This can be achieved by studying larger patient cohorts, particularly those from South Asia (populations from the Indian Subcontinent) who were long-term subjects of Portuguese, Dutch, and British colonialism may provide unique resources to investigate.

However, it is important to note that the existing literature presents differing conclusions regarding the European admixture with the populations from Indian Subcontinent. According to the findings of Reich et al. in 2009, it was determined that the Ancestral North Indians (ANI) exhibit genetic similarities with individuals from the Middle East, Central Asia, and Europe [63]. Conversely, the "Ancestral South Indians" (ASI) were found to be genetically distinct from ANI. According to a study conducted by Neus Font-Porterias et al. in 2019, it was determined that the potential ancestral group of the proto-Roma, which is the largest transnational ethnic minority in Europe, can be traced back to a Punjabi group with minimal levels of West Eurasian ancestry [64]. Furthermore, the same study has revealed the presence of a multifaceted West Eurasian element, comprising approximately 65% of the Roma population. This finding can be attributed to the intermingling that transpired between non-proto-Roma groups and the Roma community during the period spanning from 1270 to 1580. Intriguingly, a recent study conducted by Perera et al. 2021 examined the four major ethnic groups in Sri Lanka, namely Sinhalese, Sri Lankan Tamils, Indian Tamils, and Moors. The study found that all Sri Lankan ethnicities, with the exception of Indian Tamils, exhibited a close clustering with populations from the Indian Bhil tribe, Bangladesh, and Europe. This clustering pattern suggests a shared Indo-Aryan ancestry among these populations [65].

Although consanguineous marriages are infrequent in Western societies, when Middle Eastern populations are considered; Iran (consanguinity: 50.7% in urban, 86.2% in rural) [66] Riyadh from Saudi Arabia (Consanguinity 80.6% in Samtah and 62.8% in Riyadh) [35] shows significant deletion percentages in *DMD* gene reported as 80% in Iran and 78% in Riyadh correspondingly. Elhavary et al. suggest that the observed higher consanguinity rate in Riyadh may have a link with the increased *DMD* deletion rates (77.8%) observed in Riyadh [58, 67]. Moreover, Algeria, a country in Northern Africa, has reported a higher rate of consanguinity (36.6%) [68]. Selvaciti et al. reported a noteworthy finding that Algerian patients exhibit a higher percentage (77%) of DMD deletions compared to Eastern Europeans [57], whose mutations are primarily nonsense (31%) followed by deletions (29%). Notably consanguinity account for 20–50% of marriages in various parts of Africa and Asia, particularly in South Asia [69]. In Pakistan and the southern portions of India, consanguineous marriages account for around 70% and 23% of all marriages, respectively [70]. In this context, it is possible to infer that consanguinity may have played a role in the higher rates of *DMD* gene deletions observed in South Asians, Africans, and Middle Eastern countries. However, it is important to note that while consanguineous unions can result in a higher occurrence of autosomal recessive disorders, there is ample evidence to suggest that consanguinity does not elevate the risk for autosomal dominant conditions or X-linked recessive conditions [71–77]. The available scientific evidence does not provide a strong basis for linking the higher rates of *DMD* gene deletions observed in South Asians, Africans, and Middle Eastern countries to consanguinity.

In this context, the increased frequencies of deletions in the DMD gene may be attributed to various mechanisms that are involved in the formation of genomic rearrangements [78–80]. Non-allelic homologous recombination (NAHR) is an important mechanism that can explain the frequencies of deletions and duplications [81–83]. The NAHR mechanism, specifically the crosslinking of Alu repeats, has been implicated as a causal factor in deletions affecting various genes, including the DMD gene [84]. However, it is important to note that if NAHR caused both deletions and duplications, it would anticipate comparable frequencies of deletions and duplications for each intron. However, this is not observed in the case of DMD [38]. Thereby it is reported that nonrecurrent events typically do not arise through NAHR. Instead, nonhomologous end joining (NHEJ), which involves the ligation of double-strand breaks, is often suggested as a mechanism for nonrecurrent intragenic deletions and duplications [85]. Several studies [86–90] have provided supporting evidence for this in DMD through the sequencing of deletion breakpoint junctions in the *DMD* gene. Moreover, it has also been proposed that duplications may occur at various stages of the cellular cycle. Similar to point mutations, deletions are primarily inherited from the maternal lineage, whereas duplications are passed down through the paternal germ line [91–93].

When the variation hotspots of our cohort were compared to the information available in the literature (Fig. 2), distinct distal hotspot has been identified for Netherlands, which ranged from (exon 8–61). This observed variation may have been influenced by clustering, which is connected to locally constrained gene flow across significant Dutch rivers and to country-wide ancestry gradients from neighboring territories [94]. For duplications ranging from (exon 50–79) and (exon 45–50), respectively, Iran from the Middle East, and, the African region stood out as unique hotspots. The observed uniqueness in duplication hotspots in Iran and Africa may be due to the high consanguinity rates associated with these populations.

The higher deletion frequency in the distal hotspot region (Fig. 1) and low duplication frequency observed in South Asians may provide insight into the feasibility of implementing conventional molecular diagnostic approaches such as mPCR, which can easily detect about 90% of the deletions in the hotspot region. Thus, it is proposed to develop tailored molecular diagnostic algorithms that are regional and population-specific and easily implemented in low resource settings. [95].

### Delay in onset of the symptoms to molecular diagnosis

It is notable that diagnostic delays persist in traditionally disadvantaged groups, such as patients from developing countries and with lower socioeconomic status, because access to subspecialty care and genetic testing is difficult for patients from developing countries [7], including Sri Lanka [6]. (Fig. 4).

It is noteworthy that the average age of patients receiving their first clinical evaluation in our cohort was four years (Table 1), the same as the age of onset of symptoms. This is in contrast to data reported in India [18]; [Age at symptom onset—(3.7 ± 1.9 years), Age at first clinical evaluation – (8.1 ± 2.5 years)], China [104]; [Age at symptom onset- (3 years), Age at first clinical evaluation- (6–8 years)] Saudi Arabia[58]; [Age at symptom onset- (1–3 years), Age at first clinical evaluation- (9–12 years)]. Despite the fact that the average age of symptom onset in our patient cohort was 4 years (Table 1), when the first clinical examination was performed, only 21% of patients (29/138) were referred for molecular diagnostics before the age of 5 years. In our cohort, this has increased the average age of referral to molecular diagnostics to 7.8 years, indicating a delay in receiving an accurate diagnosis.

The observed delay may be attributable to the following: (1) The time required for referral to a specialist (Neurologist/ Pediatric Neurologist) and the difficulty in obtaining access to crucial diagnostic tests that must be performed at a government tertiary care hospital, (2) Lack of awareness among clinicians regarding the significance of molecular diagnostics as the gold standard for DMD confirmation; and (3) Neurogenetic testing is almost nonexistent in Sri Lankan government hospitals and only available at exorbitant costs in a few private sector centers. To the best of our knowledge, the neuromolecular diagnostic service established by the corresponding author at a government institute is the first of its kind to offer free molecular diagnostics for certain neuromuscular and neurodegenerative diseases. Moreover, CPK screening remains as the initial approach in testing for muscular dystrophies in resource limited settings where molecular diagnosis is not frequently available at a reduced cost. In our study, the majority of patients in our patient cohort underwent CPK evaluation during their initial visit to the healthcare professional (Table 1). This evaluation prompted their enrollment in the molecular diagnostic program, which provides genetic confirmation at no cost. The authors have previously addressed the relationship between age, mutation pattern and CPK levels in this patient cohort in a comprehensive manner, as documented in Wijekoon et al., 2023 [105]. Therefore, further discussion on this topic will not be included in the present paper. Thus CPK screening may be suggested in primary care as an approach in suspected early diagnosis of dystrophinopathy in resource limited settings which should be followed by a confirmatory molecular diagnostic approach, which will further reduce the diagnostic delay.

Despite being one of the most comprehensive studies conducted to date on dystrophinopathies in Sri Lanka, we acknowledge the following limitations in our study. *N* = 87 patients were negative for MLPA analysis; however, due to limited infrastructure and financial constraints, we could not perform genome sequencing for the MLPA negative cases, which we open up for future international collaboration. In addition, carrier detection for the mothers and female siblings of the probands was only conducted in a limited number of cases at the request of the consultant neurologist/pediatric neurologist due to the lack of genetic counselling services within the Sri Lankan health care system.

### DMD gene variations interpretation from genetic report to clinic and the reading frame rule

It is important to remember that false positive results in MLPA can occur due to failed primer/probe binding, especially in single exon deletions. In this regard, according to Kim et al. 2016, MLPA has been found to have a false positive rate of approximately 15% in cases of large gene rearrangements that affect a single exon [31]. In addition, Buitrago and colleagues documented a 40% rate of false-positive results among individuals who were identified as having mutations affecting single exons through MLPA testing [32]. Hence, it is recommended that medical professionals in the clinic take into account the variation detection procedure utilized before drawing any conclusions about a single exon deletion [9]. The European Molecular Quality Genetics Network's (EMQN) best practice recommendations for genetic testing in dystrophinopathies include reconfirming single exon deletions discovered in MLPA by PCR [2]. In this analysis, we found *n* = 28 (20%) DMD patients with single exon deletions, with exon 44 and exon 51 being the most commonly deleted single exons (Table 2). Following EMQN protocols, all single exon deletions were re-confirmed by Multiplex PCR before being reported to the clinic.

To predict potential Duchenne or Becker effects of the deletion or duplication discovered using DNA data, the "reading frame rule" has gained popularity [8]. However, since deviations to the reading frame rules are frequent, the predictive sensitivity of the "reading frame rule" has been questioned. It can be difficult for medical practitioners to judge whether to classify a patient as having Duchenne or mild to moderate Becker by merely interpreting the reading frame, since "leaky" variations that are initially out-of-frame are found to produce low quantities of dystrophin, which will reduce the disease severity by 3–4% [33].

As presented in Table 2, in our population, a total of 17 out of 149 mutations, representing 11.4%, were found to be non-compliant with the reading frame rule. The comparative analysis of our value revealed that it is higher than the percentages reported in various regions including Tamil Nadu, India (3.9%) [29], Bangalore, India (8.4%)[34], Saudi Arabia (5.6%) [35], France (4%) [36], Italy (5.4%) [37], Brazil (9.6%) [38], TREAT-NMD DMD Global database (7%) [9], UMD-DMD database (4%) [36], and Leiden database (9%) [39]. However, our value is lower than the values reported in China (13.6%) [40] and Spain (15%) [41]. Mateu et al. [38] report that deletions exhibit a relatively low number of exceptions to the reading frame rule, whereas duplications and point mutations tend to have a greater probability of exceptions to the reading frame. In contrast, our cohort exhibited a reading frame exception rate of 8.7% for deletions and 2.6% for duplications. Nonetheless, the analysis of point mutations was not feasible in the present investigation, a constraint that we duly recognize.

When in-frame variations are evaluated further, it is reported in the literature that in-frame variations encoded by exons 64–70, 2–10, and 32–35 are associated with a DMD phenotype, as in-frame variations bordering the aforementioned regions will not produce a functional dystrophin protein [42]. Contrarily, 94% (16/17) of the in frame DMD patients in our dataset had a variational hotspot between exons 45 and 60 (Table 2). Consequently, it is suggested that the in-frame variational hotspot (exon 45–60) found in our study may represent a novel population-specific in-frame hotspot that has to be further studied in regional patient pools and validated via dystrophin protein levels.

In keeping with a previous study by Yan-Li Ma et al. 2022 [43] that revealed a predictive sensitivity of 86.8% for DMD, our cohort's predictive sensitivity for DMD based on the frame-shift theory was 85% (117/138) (Table 2). It is interesting to note that early gross motor development milestone delay is documented in the literature as a clinical feature of DMD but not BMD [44]. However, it is noteworthy that the gross motor development milestone delay, when taken alone has a limited ability to predict DMD, particularly in situations when the disease has in-frame variations [43].

Yan-Li Ma et al. 2022 [43] reported that, the reading-frame rule combined with the walking alone milestone significantly improved the early diagnosis rate of DMD, particularly the cases with in-frame variations, with a diagnostic coincidence rate increased to 93.49%, significantly higher than that predicted by reading-frame rule alone (*P* = 0.05). In this context, 15/17 (88%) of in-frame DMD cases in our study showed global development delay, of which, language delay accounting for 11/15 (73%) and motor development delay accounting for 13/15 (87%) of these cases, respectively (Table 2). It's interesting to note that none of the BMD patients in our dataset exhibit a generalized developmental delay (Table 1). Our findings thus provide more evidence in favor of the idea that the reading-frame rule should be combined with both language delay and motor development delay to increase the prediction sensitivity of DMD, particularly in situations when in-frame variations are present.

## Conclusion

The largest and most well-established DMD mutation database in Sri Lanka demonstrates DMD gene deletions and duplications that are primarily concentrated in exons 45–55 and 2–20, respectively, which are consistent with the globally observed variation hotspots. Importantly, a unique, distinct mutation pattern of exon 45–60 was identified as a novel in-frame variation hotspot, which would contribute in personalized medicine to rational design of mutation-specific therapies. Furthermore, we have observed intriguing disparities in deletion and duplication frequencies when comparing our data to other Asian and Western populations.The utilization of mPCR as an initial molecular diagnostic method is considered highly feasible for countries with limited resources, owing to its 95% detection rate for deletions as identified in our study. Thereby, the authors propose an initial screening method using mPCR, then an assessment of cases that test negative for mPCR and have ambiguous mutation borders using MLPA. Our findings may have important implications in the early identification of DMD with limited resources in Sri Lanka and to develop tailored molecular diagnostic algorithms that are regional and population-specific and easily implemented in resource limited settings.

### Supplementary Information

**Additional file 1: Table S1.** Additional mutations and deletion borders identified by MLPA over Multiplex PCR.

## Data Availability

The datasets used and/or analysed during the current study are available from the corresponding author on reasonable request.

## Abbreviations

DMD: Duchenne muscular dystrophy
BMD: Becker muscular dystrophy
MLPA: Multiplex Ligation Dependent Probe Amplification
mPCR: Multiplex PCR
FSIQ: Full Scale IQ
WISC-IV: Wechsler Intelligence Scale for Children
