# Supplementary material for: Title-molecular diagnostics of dystrophinopathies in Sri Lanka towards phenotype predictions: an insight from a South Asian resource limited setting
Source: Eur J Med Res. 2024 Jan 9;29:37. doi: 10.1186/s40001-023-01600-x (PMC10775540; doi:10.1186/s40001-023-01600-x)
Supplement: Supplementary file 1 — Additional file 1: Table S1. Additional mutations and deletion borders identified by MLPA over Multiplex PCR. [file 40001_2023_1600_MOESM1_ESM.docx]

**Table 01- Additional mutations and deletion borders identified by MLPA over Multiplex PCR**

| Sample Number | Case ID | mPCR Result | MLPA Result |
| --- | --- | --- | --- |
| 01 | DMD005 | Exon 47-52 deleted | Exon 46-52 deleted |
| 02 | DMD029 | Exon 47 deleted | Exon 46-47 deleted |
| 03 | DMD033 | Exon 47 deleted | Exon 46-47 deleted |
| 04 | DMD034 | Exon 8-19 deleted | Exon 8-25 deleted |
| 05 | DMD035 | Exon 47-50 deleted | Exon 46-50 deleted |
| 06 | DMD040 | Exon 51-53 deleted | Exon 51-55 deleted |
| 07 | DMD042 | Exon 47-51 deleted | Exon 46-51 deleted |
| 08 | DMD044 | Exon 47 deleted | Exon 46-47 deleted |
| 09 | DMD049 | Exon 47-53 deleted | Exon 46-53 deleted |
| 10 | DMD055 | Exon 47 deleted | Exon 46-47 deleted |
| 11 | DMD056 | Exon 45-53 deleted | Exon 45-54 deleted |
| 12 | DMD064 | Exon 8 deleted | Exon 8-11 deleted |
| 13 | DMD065 | Exon 3-8 deleted | Exon 3-11 deleted |
| 14 | DMD066 | Exon 1-19 deleted | Exon 1-42 deleted |
| 15 | DMD067 | Exon 1-19 deleted | Exon 1-42 deleted |
| 16 | DMD069 | Exon 3-6 deleted | Exon 3-7 deleted |
| 17 | DMD073 | Exon 19-44 deleted | Exon 18-44 deleted |
| 18 | DMD080 | Exon 8 deleted | Exon 8-10 deleted |
| 19 | DMD095 | Exon 47-48 deleted | Exon 46-48 deleted |
| 20 | DMD109 | Exon 3-19 deleted | Exon 2-29 deleted |
| 21 | DMD111 | Exon 3-6 deleted | Exon 3-7 deleted |
| 22 | DMD112 | Exon 48-53 deleted | Exon 48-54 deleted |
| 23 | DMD114 | Exon 47-52 deleted | Exon 46-52 deleted |
| 24 | DMD115 | Exon 47-48 deleted | Exon 46-48 deleted |
| 25 | DMD118 | Exon 45 deleted | Exon 45-46 deleted |
| 26 | DMD121 | Exon 8 deleted | Exon 8-11 deleted |
| 27 | DMD128 | Exon 43 deleted | Exon 38-43 deleted |
| 28 | DMD129 | Exon 3-43 deleted | Exon 1-44 deleted |
| 29 | DMD130 | Exon 48-53 deleted | Exon 48-54 deleted |
| 30 | DMD133 | Exon 47-49 deleted | Exon 46-49 deleted |
| 31 | DMD 134 | Exon 43-45 deleted | Exon 31-45 deleted |
| 32 | DMD024 | No deletion | Exon 61-62 deleted |
| 33 | DMD025 | No deletion | Exon 61-62 deleted |
| 34 | DMD047 | No deletion | Exon 64-67 deleted |
| 35 | DMD048 | No deletion | Exon 64-67 deleted |
| 36 | DMD072 | No deletion | Exon 20-42 deleted |
| Duplications identified by MLPA over mPCR | | | |
| 01 | DMD001 | No deletion | Exon 7-9 duplicated |
| 02 | DMD002 | No deletion | Exon 6 duplicated |
| 03 | DMD021 | No deletion | Exon 52 & 60 duplicated |
| 04 | DMD022 | No deletion | Exon 53 duplication |
| 05 | DMD050 | No deletion | Exon 52-67 duplicated |
| 06 | DMD057 | No deletion | Exon 8,9 & 11 duplicated |
| 07 | DMD086 | No deletion | Exon 35-43 duplicated |
| 08 | DMD105 | No deletion | Exon 8-9 duplicated |
| 09 | DMD122 | No deletion | Exon 2-7 duplicated |
| 10 | DMD124 | No deletion | Exon 6 duplicated |
| 11 | DMD127 | No deletion | Exon 14-17 duplicated |
| 12 | DMD132 | No deletion | Exon 49 duplicated |
